# Psychometric properties of the Marschak Interaction Method of Psychometrics and the Assessment of Parent–Child Interaction within residential care and non-referred settings

**DOI:** 10.3389/fpsyg.2023.1296113

**Published:** 2024-01-08

**Authors:** Stine L. Jacobsen, Susan Hart, Jens Anderson-Ingstrup, Gustavo Gattino

**Affiliations:** Music Therapy, Art, Aesthetics and Health, Department of Communication and Psychology, Aalborg University, Aalborg, Denmark

**Keywords:** caregiver-child, assessment, observation-based, interaction-based, nonverbal communication, emotional communication

## Abstract

**Introduction:**

Assessment and identification of children with developmental needs and their interaction with primary caregivers are critical for emotional and social development. However, to the best of our knowledge, there is a scarcity of valid observation-based tools that guide the work with family communication, which is essential for the child’s healthy development.

**Method:**

The Marschak Interaction Method of Psychometrics (MIM-P) and Assessment of Parent–Child Interaction (APCI) are both interaction and observation-based assessment tool, and they were explored for their validity and reliability in assessing caregiver-child interaction. The study included 30 trained and certified professionals who recruited referred and non-referred caregiver-child dyads over 11 months. Assessment data was collected from 139 caregiver-child dyads for the MIM-P with 278 individuals (100 referred and 178 non-referred) and 129 caregiver-child dyads for the APCI with 257 individuals (95 referred and 162 non-referred).

**Results:**

The psychometric analyses show that both the MIM-P and APCI presents relevant sources of reliability and validity for assessing caregiver-child interaction including interrater reliability, internal consistency, test re-test reliability as well as concurrent and construct validity.

**Discussion and conclusion:**

The study highlights the need for observation-based assessment tools within social work and contributes to the understanding of the importance of relationships and interaction in children’s emotional and social development. However, further research is needed to explore norms and further strengthen implementation and quality of the tools.

## 1 Introduction

The referral of children to mental health institutions due to regulatory problems and attachment issues has become a significant concern in recent times. It highlights the importance of the complex and bi-directional link between caregiver-child synchrony, the healthy development of emotional regulation, and the need to assess both the child and the caregiver-child interaction or relationship (Bowlby, 1953a,b; Trevarthen, 2005). Colegrove and Havivhurst (2016) highlighted the lack of observational tools and interventions that focus on non-verbal communication in parent–child dyads, although previous and recent research has emphasized the importance of professionals understanding the nonverbal and emotional dynamics of vulnerable families and caregiver-child interaction (Colegrove and Havivhurst, 2016; Apter et al., 2020).

Research supports the shift toward relationship-focused assessment and interventions, with the closest caregiving relationships providing a more accurate predictor for future outcomes than an examination of a child’s individual characteristics (Shonkoff et al., 2012). Parental sensitivity, which includes the ability to structure and support a child in their zone of proximal development, has enduring implications for development and adaptation into adolescence and adulthood (Grossmann et al., 2006; Sroufe et al., 2010).

In 2017, one of the authors conducted an unpublished literature review focused on dyadic assessment methods for caregiver-child interaction within the age range of 3–12 years (Hart, 2018). The ensuing Table 1 encapsulates the findings derived from this examination.

**Table 1:** Dyadic assessment methods for caregiver-child interaction.

Name of test	Author, year	Age group	Main focus	Analysis method
EAS (Emotional Availability Scale)	Biringen and Easterbrooks (2012)	0–16 years	Observation of nonverbal communication in the parent–child dyad, derived from attachment theory.	Video/rating
IFIRS (Iowa Family Interaction Rating Scales)	Melby and Conger (2001)	5–12 years	Parents and child are instructed to engage in a discussion and problem-solve issues. Use of silence, smiles, hugs, pauses in discussion, body position. Draws on social interaction, behavioral and social contextual theory.	Video/rating
Kahen Affect Coding System.	Gottman et al. (1996)	11–16 years	Emotion socialization. Vocal tone, facial expression, posture, gesture. Draws on social interaction, behavioral and social contextual theory.	Video/rating
SACS (Simple Affect Coding System)	Jabson et al. (2003)	All ages	Objective display of affect as relationship interaction. Interpersonal space, facial expression, tone of voice. Draws on social interaction, behavioral and social contextual theory.	Video/rating
SCIFF (System for Coding Interactions and Family Functioning)	Lindahl and Malik (2000)	0–16 years	Parents and child are instructed to discuss a recent family argument, while the interaction is rated on a Likert-type scale. Derived from a systemic approach.	Video/rating
SPAFF (Specific Affect Coding System)	Gottman and Krokoff (1989)	All ages	Coding interactions through facial expression, voice pitch, volume and tempo, posture, gesture. Draws on social interaction, behavioral and social contextual theory.	Video/rating

This literature review identified six assessment methods, categorized into three overarching theoretical frameworks. Specifically, only one other method (besides APCI & MIM-P) is grounded in attachment theory, two in a systemic approach, and three in communication theory. All the identified methods are rooted in observation techniques, developed between the late 1990s and 2015, relying on structured or unstructured observations of video recordings. These observations are subsequently rated either qualitatively or quantitatively, based on video excerpts.

The six assessment methods predominantly focus on nonverbal communication, objectively rated through the detection of facial expression, voice pitch, volume and tempo, posture, and gesture. Alternatively, through subjective psychological values, defined as dimensions such as parental sensitivity, structuring, engagement, child responsiveness, and involvement. Five of these methods utilize a Likert-type scale or a multi-modal tool for rating responses. The three attachment theory-based assessment methods for parent–child interaction are the EAS, APCI, and MIM-P and they all have a clear understanding that the parent–child relationship is an asymmetrical relationship, and that the parents play a crucial role and bear the responsibility for creating a setting where the child feels comfortable and regulated.

Emotional Availability Scale (EA; Biringen and Easterbrooks, 2012) theory analyzes the parent–child relationship emphasizing emotionality. EAS comprises parental sensitivity, structuring, non-hostility, and non-intrusiveness and from the child’s side responsiveness and involvement. EAS refers to the degree to which a connection is genuinely affectively positive and to the extent to which the dyad can accommodate and downregulate negative affect also keeping in mind, that these regulative needs of the child change during development. As it uses an unstructured setting EA observation have varied from stressful separation-reunion contexts into most used free-play situations videotaped either at the clinic of at home for a minimum of 20 min. All the dimensions are rated top-down as global perception as well as from bottom-up requiring rating of all six dimensions on a 29-point metric. Extant publications on the EAS have shown that both parent and child dimensions of EAS relate to key aspects of the mother–child relationships as well as to maternal characteristics and child behavior, and certain risk in developmental psychopathology (see Bornstein et al., 2012). Compared to APCI and MIM-P EA measures different aspect of the dyadic relationship and use a different kind of setting.

MIM-P is a structured play-based observational method that has the dyadic relationship as its field of investigation. The building blocks for the original MIM were laid out by Marianne Marschak, who in 1958 developed MIM under the auspices of the Yale Child Study Center. Marschak’s original MIM model was called the Controlled Interaction Schedule (CIS), and several articles were published on the model under that name before it was changed in the late 1960s to the Marschak Interaction Method (Booth et al., 2011). Marschak published her first MIM design in 1960, and at Michael Reese Hospital in Chicago, her daughter, Ann Jernberg, and her colleague Austin DesLauriers first used MIM in long-term studies in 1964 (Booth et al., 2011). When Jernberg became responsible for the psychological services of the newly established Head Start Program in Chicago in 1967, she made use of MIM in relation to vulnerable children and their mothers. In this context, Marschak made the first film recording of the use of MIM in practice (Marschak, 1967). Marschak wanted to capture the interaction between parent and child. For MIM use, Marschak therefore selected only material that could be expected to capture dimensions of interaction behavior between the adult and the child, so that the quality of both adult and child behavior could be examined (Booth et al., 2011). Over the years, MIM became increasingly integrated with the intervention method Theraplay, and long before Marianne Marschak died, she had accepted that Jernberg would adapt MIM to Theraplay.

MIM was originally developed as a qualitative clinical tool or a qualitative observation method. Since the 1990s, several diverse groups have attempted to standardize the method (see, e.g., Hitchcock et al., 2008; Martin et al., 2008; Bojanowski and Ammen, 2011; Salo and Mäkelä, 2018). In Denmark, we have come one step closer to further development of MIM with the development of a scoring system with a theoretical anchoring in neuroaffective developmental psychology. Thus, the observation method is both anchored in an attachment theory context and made quantifiable through psychometric qualities (hence MIM-Psychometric), so that it can be included in a research study where it becomes possible to conduct reliability and validity studies of the test.

Almost 20 years ago in 2005, the first author of this manuscript embarked on her professional journey as a music therapy intern within a family care center situated in Denmark. She investigated the feasibility of evaluating parent–child interactions as a music therapist within a multidisciplinary team. The family care center was renowned as an alternative approach to safeguarding children from being separated from their parents. Upon arrival, many of the families were often frustrated and anxious, leading them to deviate from their customary behavior. Some families presented a facade, concealing their genuine emotions, while others found themselves overwhelmed by their anxiety, rendering them unable to display their usual strengths.

Despite challenges, music therapy positively influenced families, allowing them to relax and connect. Jacobsen developed an initial qualitative version of APCI during her master’s thesis, collaborating with Professor Tony Wigram in 2007 (Jacobsen and Wigram, 2007). Recognizing its effectiveness, Jacobsen pursued a Ph.D. to enhance the tool through quantitative methods, aiming for rigorous validation. Her motivation was to offer objective assessments for families, particularly those with emotionally neglected children and struggling parents. Jacobsen was committed to ensuring decisions about removing a child from their family were based on objective measures and professional evaluations instead individual subjective interpretations (Jacobsen et al., 2014; Jacobsen and Killén, 2015; Jacobsen and McKinney, 2015).

Only a few assessment methods/tests in the literature review revealed video-based observations of communication and focused on developing and examining psychometric properties. MIM-P has been the focus of detailed examination, resulting in the development of two distinct scoring systems by separate researchers. Pilot psychometric studies have been conducted for both scoring systems: the Emotional Interaction Style (EIS) devised by Salo (Rye and Drozd, 2021) and MIM-P devised by Hart (2018). These preliminary investigations aim to establish the reliability and validity of the respective scoring systems. In a parallel, APCI underwent psychometric scrutiny in 2015, contributing to the broader understanding of its measurement properties (Jacobsen and McKinney, 2015). Similarly, the EAS has been subjected to comprehensive investigations into its validity and reliability. The extensive scrutiny of EAS is reflected in studies conducted by Aran et al. (2022), Salo and Flykt (2010), as well as Salo et al. (2009), collectively contributing to the robustness of its psychometric foundation. Historically and even currently, a young child’s functioning is often assessed outside of the context of their relational environment or without representing a child’s functioning with reference to regulatory dynamics between caregiver and child (Dickson and Kronenberg, 2011; Boele et al., 2019).

The child’s personality development and the development of self-regulation skills are supported by implicit synchronization processes linked to coordinated interactions, in which small moments of encounters occur between the child and the caregiver (Trevarthen, 1993, 2005; Stern, 2000). These are structured and synchronized interactions that can be assessed and measured through MIM-P based on structured interaction activities, where the assessor captures the interaction between the caregiver and child. The purpose is to uncover both the child’s development and developmental processes in the child’s relational environment to find the “key” to relevant goals and interventions aiming to develop the child’s emotional, personality and social skills and support the child’s relational environment (Marschak, 1960; Salo and Mäkelä, 2018).

Interpersonal interaction depends on non-verbal communication channels. Verbal language is an inadequate medium to express the quality, intensity, and nuances of emotions and affect in different social situations (Mandal and Ambady, 2004). To understand verbal and nonverbal communication, Knapp and Hall (2009) argued that the ability to send and receive nonverbal messages is an important part of communication competence. For parents to attune to their child, they must be able to decode non-verbal cues and respond sensitively to expressed needs. Nonverbal communication skills are crucial for parents, as emotional parenting is about providing predictable and accessible emotional communication; something that is strongly influenced by parents’ relational competence (Fonagy and Target, 1997). It is the establishment or re-establishment of the pre-verbal ability for rhythm and synchronization between child and caregiver that can be explored through improvisations and through relational focus. A non-verbal and musical approach can be particularly valuable when working with families or dyads where the level of mentalization is not within reach or not part of the zone of proximal development yet (Hart, 2016).

Hence, there is a growing need for tools which are standardized and present sources of validity for assessing caregiver-child interaction that are useful in planning functional and relationship-based intervention. This article aims to present a study investigating the psychometric properties of observation-based tools and the importance of assessing the relational environment as part of assessing the child’s social, emotional, and personality capacity with methods that are structured and presents sources of validity (Hart, 2018; Hart and Jacobsen, 2018).

The research questions addressed in this article consist of the following:

- What are the psychometric properties of the MIM-P including reliability and validity of the scale and subscales: Structure, Co-regulation, Engagement, Nurture, and Challenge?
- What are the psychometric properties of the APCI including reliability and validity of the scale and subscales: Mutual Attunement; Nonverbal Communication, Emotional Support; Parent–Child Interaction and APCI Profile?

## 2 Materials and methods

The study is part of a larger collaboration between Aalborg University and ‘LIVSVÆRK’, a voluntary Danish association that since 1898 has provided social support for people in vulnerable positions through professionally qualified services. The larger research study included training 110 professionals in four newly developed assessment tools focusing on emotional and social skills and collecting assessment data from 864 participating children, adolescents, and adults. The tools implemented were Neuroaffective Analysis (NAA), Emotional Mentalizing Scale (EMS), and the two interaction and observation-based tools relevant for this partial study was The Marschak Interaction Method of Psychometrics (MIM-P) and Assessment of Parent–Child Interaction (APCI). In this collaboration with LIVSVÆRK, the assessment methods are meant to be used as a framework for providing background knowledge to offer realistic interventions targeting the interaction between a caregiver and child. Results from psychometric analyses of NAA and EMS are presented through other submitted but not yet published articles.

The psychometric properties of MIM-P and APCI are the focus for the current study, and they are explored for their validity and reliability in assessing caregiver-child interaction. The study includes 30 trained and certified professionals who rated 139 dyads using MIM-P with a total of 278 individuals (100 referred and 178 non-referred) and 129 caregiver-child dyads for the APCI with a total of 257 individuals (95 referred and 162 non-referred).

### 2.1 Project design and organization

A key objective of the research project was to implement two out of four assessment methods and collect data from daily practice to further validate the assessment methods and examine their psychometric properties. This was done through three phases planned together with the participating professionals and residential care institutions to ensure data collection and ethics. In the first 6 months phase, assessment training courses were conducted to ensure quality in the implementation. The training courses for the different methods ranged from 20 to 30 participating professionals and consisted of 3 days’ training with a subsequent online certification. Around 80 percent managed certification through distinct types of online testing and try-outs with non-referred dyads.

During the following 12 months and the second phase, the certified professionals collected data and analyzed each other’s data. Looking at the observation-based interaction assessment tools of MIM-P and APCI, 201 dyads participated in recruitment and data collection as 30 trained and certified professionals performed the observation-based assessment sessions and rated the video data using online web-based platforms. The third and final phase focused on data analysis, reporting, dissemination, and further practice implementation.

### 2.2 Participants

In organizing the study and recruiting professionals and participants some specific considerations were made. The professionals were required to have a basic education as a pedagogue, psychologist, social worker or other relevant education at BA or MA level to ensure quality and comparability. Furthermore, the professionals were organized in teams with 2–4 trained professionals from each institution, as they followed each other during the course participation to ensure continuous supervision and implementation quality. The team helped each other in the use and understanding of the assessment methods. It was possible for each team to be formed across institutions to support cohesion. Specifically, they had the task to analyze each other’s data as part of the investigation of the reliability and validity of the methods. The institutions took responsibility to ensure data collection and researchers ensured that storage of data was carried out in accordance with the General Data Protection Regulation (GDPR), which enabled the professionals to focus their time on training and data collection. The study only included anonymized data, which was submitted through customized Excel files. Thus, the research project did not include any video files or documents with the names of participating caregivers or children.

The referred participants were all referred to one of LIVSVÆRK’s residential care institutions and the main reason for referral was a concern for the child’s mental health and/or concern for the parent’s capacity to support the child’s development. The non-referred participants were recruited individually by professionals from invitations at local schools and within the local area community with an exclusion criteria of the family having no overt contact with social services, no developmental disabilities or no psychiatric diagnosis.

#### 2.2.1 Participant demographics

As mentioned, this article refers to a partial study where MIM-P data included 26 professionals from seven different residential care institutions and the APCI data included 21 professionals from five residential care institutions. Out of these 47 professionals, 17 were certified in and collected data using both MIM-P and APCI. The MIM-P analyses included 139 recruited dyads and the APCI included 129 dyads, and this formed the basis for the psychometric investigations and analyses of reliability and construct validity. Included in the MIM-P were 37 dyads of professional caregivers and referred children; 28 referred parents and referred children; 67 non-referred parents and non-referred children; 7 non-referred parents and referred children. Included in the APCI were 37 days (about 1 month 6 and a half days) of professional caregivers and referred children; 29 referred parents and referred children; 63 non-referred parents and non-referred children. As the professionals oversaw data collection and rating sessions as well as oversaw finding participants, it was not possible to blind the groups of non-referred and referred.

### 2.3 Instruments

#### 2.3.1 MIM-P

MIM-P is used for parental competence examinations and child psychological examinations. As the interpretation of MIM-P is based on clinical insight and in-depth knowledge of the child’s development and the interaction between caregiver and child, professionals using MIM-P must have extensive clinical experience. An important aspect of the use of MIM-P lies in the way in which they can provide information that strengthens the design of an intervention plan. When caregiver and child are together and carry out the specific activities included in the method, typical interaction patterns emerge. Many interaction patterns are not conscious, which is why observing interaction firsthand can nuance the parents’ and child’s stories about themselves and their family. For example, watching problem behavior unfold in the interaction and observing how it occurs can provide better insight into how it can be changed. By seeing strengths and coping strategies, one gains an insight into the resources that also exist in family dynamics (Booth et al., 2011). For inclusion purposes or in school and treatment homes, MIM and MIM-P can be used advantageously when finding the child’s development potential in contact and interaction with the primary educator.

MIM-P consists of a MIM-P suitcase with 10 numbered bags. In addition, MIM-P consists of 10 activity cards (see Supplementary material). Each activity card is placed in the bag, together with the material required for several of the activities. When the caregiver and child are about to start, they are instructed to sit next to each other at a table with a video camera opposite. They are instructed that there is no fixed amount of time for carrying out the activities, but most spend approx. 30–45 min. Once finished, they call the assessor, who asks the caregiver and child some questions regarding the MIM-P activities. The MIM-P activity cards are available in three versions: Children 0–2 years, Children/adolescents 3–17 years, and a Family version. The method is based on 10 simple structured activities that caregiver/parent and child perform together. MIM-P in this study focuses on children from 3 to 17 years together with their primary caregiver (see list of activities in Supplementary material). The activities in MIM-P are designed to clarify behavior within five dimensions of caregiver and child interaction: Structure, Co-regulation, Engagement, Nurture, and Challenge (see Supplementary material). Through these dimensions, MIM-P assesses the caregiver’s ability to support the child’s emotional development and the child’s ability to accept what the caregiver offers. To uncover the dyadic interaction, an interaction score is established from multiplying the parent’s score with two, adding the child’s score and dividing the sum with three. This is to say that the caregiver bears the main responsibility of the interaction. The process is video recorded while the caregiver and child are in the room on their own. When the video recording is finished the MIM-P facilitator enters the room and asks the caregiver and child to answer a few structured questions regarding the video recording session.

#### 2.3.2 MIM-P scores

MIM-P consists of a quantitative psychometric scale to score the five dimensions; Structure (10–90), Co-regulation (10–90), Engagement (4–36), Nurture (4–36), Challenge (4–36), and Total (32–288). The scoring system is conceptually based on a thermometer with scores from 1 to 9, divided into three zones: RED, YELLOW, AND GREEN. Scores of 7–9 (green zone) indicate good and sufficient performance. A score of 4–6 (yellow zone) is less of a concern and differs most clearly from one of 1–3 in that there is potential to create change processes through intervention. A score of 1–3 (red zone) indicates concern in the dimension and indicates serious gaps in interaction (see Supplementary material).

In addition, the red and yellow zones are both divided into three; too much, unbalanced, and too little, while the green zone is undivided. This means that the thermometer is fork-shaped (see Table 2 for an example and Supplementary material). Thus, in total, three factors within each dimension are considered:

**Table 2:** MIM-P scores.

Scores	Range
Structure	10–90
Engagement	4–36
Nurture	4–36
Challenge	4–36
Co-regulation	10–90
Total score	32–288

- Sum of scores
- Number of scores in the categories red, yellow, and green
- Factors for red, and yellow categories; too much (H), too little (L) unbalanced (U).

The scoring considers the child’s development, such as the age at which the child is normally expected to be able to develop a certain competence. It considers behavior that for school children can be a sign of good socialization, such as focusing on listening to the adult, can, if it occurs on a large scale, be worrying obsessive behavior. An infant or preschooler’s search for the parent’s attention can be a healthy skill, but problematic if it is an older child. In an investigation of a relationship, MIM-P cannot stand alone, but must be supplemented with other sources of information, e.g., examination of the child’s emotional development, the caregiver’s mentalization ability, unstructured interaction observations, other people’s descriptions of the child, etc.

MIM-P requires certification to be used as an assessment tool with sources of reliability. The training course has two modules lasting 3 days and an online certification process (Hart, 2018; Hart, 2021).

#### 2.3.3 APCI

The Assessment of Parenting Competences (APCI) serves as a crucial source of quantitative data, complementing emotional and dynamic descriptions offered initially by music therapists and in this study a modified version for psychologist and pedagogues/social workers. It employs consistent, systematic instructions yielding valuable insights into family dynamics, attachment patterns, and their responsiveness to a child’s emotional needs. These scores benefit both healthcare professionals working with the family and the family itself. Remarkably, APCI demands minimal additional resources, relying on a small selection of simple musical instruments. It transforms subjective qualities of the therapeutic relationship into objective data using established and systematic methods. APCI requires certification to be used as an assessment tool with sources of reliability. The training course consists of 3 full days, analyzing 5 training dyads, and an on-line certification process (Swanick and Jacobsen, 2019).

Assessment of Parent–Child Interaction (APCI) consists of two identical 25-min assessment sessions that follow a set procedure or “protocol.” Based on the protocol, actual caregiver-child interactions can be assessed using structured and free musical activities, with analysis based on observation of improvisation and non-verbal expression. There are five specific exercises in the protocol that aim to highlight the interactions between the caregiver and child. Each assessment session is video-taped, and this is used to analyze the interactions. Scores are then calculated using a fixed analysis via a website portal. The analyses produce 16 APCI profiles that describe communication patterns and attachment behaviors (Jacobsen, 2018).

The APCI assessment protocol contains two sessions, 1 week apart, following a consistent structure. It starts with an informal opening, occasionally accompanied by a welcome song or activity, which is not analyzed. The dyad is then invited to explore musical instruments and the room freely. This initial phase assesses their reaction to an unstructured start and the primary caregiver’s spontaneous response to the child.

Next, three structured exercises follow, each with two parts. In exercise one, the dyad takes turns choosing and playing instruments, observing initiative, autonomy, and emotional responses. Exercise two involves turn-taking without talking, assessing the dynamics of sharing musical space. Exercise three focuses on following and leading events, evaluating mutual attunement and evaluating emotional responses.

Exercise four is a free play improvisation, allowing the dyad to interact without specific instructions. The facilitator joins to create a sense of safety and to gain insight into the dyad’s autonomy, relationship, and emotional responses.

Consistency is key to maintaining the protocol’s efficiency and validity. It ensures ethical trustworthiness for the dyad and establishes clear boundaries, fostering a sense of safety and trust. The APCI aims to identify concerns and positive skills within the dyad, offering hope for the future. The assessment prioritizes dyad interactions, cooperation, and engagement, with the facilitator’s role being to enable this within their defined scope (Swanick and Jacobsen, 2019).

#### 2.3.4 APCI scores

The Mutual Attunement score is derived from three of the activities in the APCI and is analyzed using a 9-point Likert scale ranging from attuned, not consistent to not attuned for the parent/caregiver and child’s leading and following behavior toward the counterpart. The Mutual Attunement score ranges from 12 to 108. See Supplementary material for more detailed information.

The Nonverbal Communication score is derived through turn-taking activities and assesses the dyad’s ability to read and produce nonverbal information. The analysis concentrates on how the parent/caregiver and child pass turns to each other, including an analysis of gestural, musical or confusing signals, and the number and quality of turns, including whether turns are interrupted. The Nonverbal Communication score ranges from 0 to 38. See Supplementary material for more detailed information.

Emotional Response Score reflects how the parent/caregiver responds to the child’s emotional needs during the assessment sessions. There are six response types derived from relevant literature in music therapy, sociology, and developmental psychology. The response types are rejecting, dominant, over-involved, passive, supportive, and emotionally exchanging. Four of the five exercises in the assessment sessions are used to collect this information. Emotional Response ranges from 0 to 16. See Supplementary material for more detailed information (Swanick and Jacobsen, 2019).

The Total APCI score is a weighted sum of the 3 sub-scores Mutual Attunement, Nonverbal Communication, and Emotional Response Score and ranges from 12 to 106. There are 16 APCI profiles which indicate a different combination of Mutual Attunement, Nonverbal Communication, Emotional Response, and Child Autonomy Behavior (which is calculated based on primary following or leading behavior in exercise 1,3, and 4). The profiles are based on the data from the primary areas of the assessment analysis. Table 3 below details APCI scores and profiles.

**Table 3:** APCI scores.

Scores	Range
Mutual attunement	7–108
Nonverbal communication skill	4–36
Emotional response	0–16
Total score	50–176
APCI profile	14–28

An example of two profile descriptions is available in Supplementary material. The APCI Profile Score is a weighted sum of the specific cutoffs for each of the sub-scores and the child autonomy behavior ranging from 4 to 28.

### 2.4 Statistical analyses

The statistical analysis focused on the MIM-P and APCI investigating analyses of interrater reliability and internal and external consistency. Construct validity was analyzed by correlating the MIM-P and APCI results between referred and non-referred groups, between gender, and between groups of professionals and parents as caregivers.

SPSS Version 29 was used in all the statistical analyses. Since the MIM-P and the APCI is a scale, and because there were sets of two professionals, the researchers chose intraclass correlations (ICC) for the interrater reliability analysis, as this estimates the extent to which data/observations are related as a function of some of shared characteristics and in this case both professionals are rating the same dyad (Cicchetti, 1994; Koch, 2006).

Cronbach’s alpha was used for analyzing the internal consistency of the MIM-P and the APCI, including an investigation of the correlation matrix between the subscales Structure, Co-regulation, Engagement, Nurture, Challenge, and Total Score for MIM-P and Mutual Attunement, Nonverbal Communication, Emotional Response, Total Score for APCI and APCI Profile (Coolican, 2014).

To further analyze the ability of the MIM-P and the APCI to differentiate between the referred and non-referred groups, a study of construct validity was chosen through an independent *t*-test. This analysis was chosen because construct validity is the scientific process of establishing that a psychological construct in fact exists or is a theoretical sound concept that fits into surrounding theory (Coolican, 2014; Furr and Heuckeroth, 2019). For the external validity between the MIM-P and the APCI, Pearson’s correlation was used. An alpha level of 0.05 was used for all statistical tests.

### 2.5 Ethical considerations

The professionals, parents, and children included in the study were informed of the study’s purpose and of the risks and value of participating. Parents signed an informed consent form and a consent form concerning the use of video recordings from the MIM-P and APCI assessment sessions. The parents and children were treated with the utmost respect and care and given as much information as possible, without overwhelming them with complex information. If any of the parents or children wanted to decline to be part of the research project, this was naturally accepted. However, no dyads chose to withdraw from the study. The Regional Committee on Health Research Ethics for Northern Jutland exempted the project from ethics approval, as the study was considered minimal risk.

## 3 Results

In the following, the study results are presented the reliability, internal consistency, and construct validity analyses.

### 3.1 MIM-P results

#### 3.1.1 Internal consistency MIM-P

The MIM-P has good internal consistency: Cronbach’s Alpha *α* = 0.822 with correlation matrix presented below in Table 4. The significant correlations between scores ranged from *r* = 0.777 to *r* = 0.980. As the scores correlate well, it seems acceptable to add all the scores to achieve a total score.

**Table 4:** Correlation matrix for MIM-P between dimensions.

MIM-P Cronbach’s alpha (*α* = 0.822) *N* = 277	Co-regulation	Engagement	Nurture	Challenge	Total
Structure	0.938**	0.881**	0.866**	0.814**	0.972**
Co-regulation		0.892**	0.907**	0.806**	0.980**
Engagement			0.844**	0.782**	0.923**
Nurture				0.775**	0.927**
Challenge					0.869**

** *p*< 0.01, *** *p* < 0.001.

#### 3.1.2 Interrater reliability MIM-P

There was a significant, positive correlation between the scoring of professional raters 1 and 2 in MIM-P on each of the five subscales and the total interaction score, which indicates strong agreement between the raters (Table 5). This suggests strong interrater reliability for the MIM-P.

**Table 5:** MIM-P interrater reliability.

MIM-P between professional 1 og 2	ICC *N* = 129
Structure	0.890***
Co-regulation	0.889***
Engagement	0.864***
Nurture	0.848***
Challenge	0.773***
Total	0.889***

*** *p* < 0.001.

#### 3.1.3 Construct validity MIM-P

In the comparison of similarities and differences between the referred and the non-referred groups, two control variables from demographic data (gender and age) were analyzed. The MIM-P sample included 44 referred and 30 non-referred boys; 28 referred and 69 non-referred girls.

Independent samples *t*-test and Chi square revealed no significant difference between the referred and non-referred groups regarding age and gender as the value of *p* is not significant (>0.05). In comparing referred and non-referred in the MIM-P groups, independent samples *t*-test revealed a significant difference between referred and non-referred regarding all subscales and total scale (Table 6). This indicates that MIM-P is equipped to differentiate between groups of referred and non-referred, which is essential in clinical work.

**Table 6:** Means and SDs between referred and non-referred in the MIM-P.

MIM-P referred/non-referred *N* = 278	Mean referred *N* = 100	SD	Mean non-referred *N* = 178	SD	df	F	t	p	95% CI
Structure	63.57	12.13	75.03	8.99	276	15.38	−8.962	0.000	[−13.97, −8.94]
Co-regulation	62.25	12.55	75.38	9.03	276	20.02	−10.073	0.000	[−15.65, −10.57]
Engagement	25.40	5.63	30.03	3.80	276	20.75	−8.169	0.000	[−5.75, −3.52]
Nurture	24.02	5.82	29.06	4.46	276	15.58	−8.089	0.000	[−6.26–3.81]
Challenge	24.73	5.06	28.81	4.40	183	1.71	−6.750	0.000	[−5.27, −2.89]
Total	200.01	39.06	238.58	28.19	276	17.91	−9.491	0.000	[−46.55, −30.56]

### 3.2 APCI results

#### 3.2.1 Internal consistency APCI

The APCI appears to have good internal consistency: Cronbach’s Alpha *α* = 0.78 with correlation matrix presented below in Table 7. The correlations between scores ranged from *r* = 0.133 to *r* = 0.803. As each score correlates well with other scores, and as they all correlate well with the APCI profile and the APCI total score, it seems acceptable to add all the scores to achieve a total score.

**Table 7:** Correlation matrix for APCI between scores.

APCI Cronbach’s alpha (*α* = 0.78) *N* = 257	Nonverbal communication	Emotional support	APCI profile	APCI total
Mutual attunement	0.387**	0.560**	0.657**	0.753**
Nonverbal communication		0.133*	0.584**	0.651**
Emotional support			0.606**	0.623**
APCI profile				0.803**

* *p*< 0.05, ** *p*< 0.01.

#### 3.2.2 Interrater reliability APCI

There was a significant, positive correlation between the scoring of professional raters 1 and 2 in APCI on each of the three subscales, the APCI profile, and the total APCI score, which indicates strong agreement between the raters (Table 8). This suggests strong interrater reliability for the APCI.

**Table 8:** Interrater reliability.

APCI between rater 1 og 2	ICC *N* = 38
Mutual attunement	0.880***
Nonverbal communication	0.751***
Emotional support	0.825***
APCI profile	0.830***
APCI total	0.605***

*** *p* < 0.001.

#### 3.2.3 Construct validity APCI

In the comparison of similarities and differences between the referred and the non-referred groups, two control variables from demographic data (gender and age) were analyzed. The APCI sample included 36 referred and 25 non-referred boys; 30 referred and 38 non-referred girls as well as 4 referred and 20 non-referred men; 25 referred and 79 non-referred women.

Independent *t*-test analyses and chi-square analyses revealed no significant difference between the referred and non-referred groups regarding age and gender. In comparing referred and non-referred APCI results, independent samples *t*-test revealed a significant difference between referred and non-referred regarding all subscales as well as total scale except Nonverbal Communication score (Table 9). This indicates that APCI is equipped to differentiate between groups of referred and non-referred, which is essential in clinical work. Further research is needed to understand subscale Nonverbal Communication, which will be discussed below.

**Table 9:** Means and SDs between referred and non-referred in the APCI.

APCI *N* = 257	Mean referred *N* = 95	SD	Mean non-referred *N* = 162	SD	df	F	t	p	95% CI
Mutual attunement	77.45	15.46	87.46	10.46	145	20.41	−6.174	0.000	[−13.20, −6.81]
Nonverbal communication	28.97	9.66	30.04	5.96	255	1.77	−1.30	0.214	[−2.75, 0.62]
Emotional support	12.57	3.65	14.48	2.17	133	61.90	−4.6	0.000	[−2.73, −1.10]
APCI profile	22.42	4.22	24.79	3.54	170	5.57	−4.61	0.000	[−3.33–1.40]
APCI total	111.08	15.59	118.52	15.06	255	2.33	−3.77	0.000	[−11.35, −3.51]

#### 3.2.4 APCI test re-test reliability

In comparing results from the identical APCI session held 1 week apart, correlation analyses using Pearson’s *r* showed significant correlations between scores as the rater was the same person. The correlations range from 0.51 to 0.85 indicating that results are similar and acceptable (Table 10). Further analysis using Paired Samples Test show no significant differences between scores from session one and session two. This indicates that APCI might be suitable for effect studies or for monitoring improvement or regression in clinical or social work.

**Table 10:** APCI test re-test reliability.

APCI between session 1 & 2	Pearsons r *N* = 55
Mutual attunement	0.800***
Nonverbal communication	0.651***
Emotional support	0.515***
APCI profile	0.683***
APCI total	0.854***

*** *p* < 0.001.

### 3.3 Correlation between the MIM-P and APCI

The external validity study included the correlation study between APCI and MIM-P with 67 individuals participating. In the calculation of correlation between MIM-P and APCI, a significant positive correlation was found on all parameters. The study used Pearson’s correlation coefficient. Alpha level of 0.05 was used for all correlations (see below in Table 11). MIM-P correlated significantly with all subscales and total scores in APCI.

**Table 11:** Correlation between APCI and MIM-P.

*N* = 63	Attunement	Non-verbal	Emotional support	Parent–child inter.	APCI profile
Structure	0.320**	0.169	0.437**	0.458**	0.499**
Co-regulation	0.321**	0.106	0.426**	0.452**	0.517**
Engagement	0.378**	0.044	0.435**	0.365**	0.451**
Nurture	0.338**	0.180*	0.417**	0.400**	0.475**
Challenge	0.231*	0.246*	0.421*	0.419**	0.512**
Total	0.335*	0.156	0.451**	0.442**	0.519**

* *p*< 0.05, ** *p*< 0.01.

These results reveal a high degree of correlation between the APCI and the MIM-P which indicate that the two assessment tools are both measuring a dyadic caregiver-child capacity, thus showing that the two tests measure various aspects, while supporting and supplementing each other meaningfully.

## 4 Discussion

The following section discusses the findings related to the reliability and validity, connection with existing literature clinical applicability, limitations, and future research.

### 4.1 Psychometric results

The analysis of interrater reliability and internal consistency revealed acceptable and good psychometrics for both the MIM-P and APCI with a few exceptions. A comparison of MIM-P and APCI scores for referred versus non-referred groups showed significant differences between the whole group of referred and non-referred. This indicates that MIM-P and APCI can distinguish between referred and non-referred groups and is in line with theories of how the focus on implicit synchronization interactions and the child’s relational environment is important for the child’s emotional and social skills and well-being and that nonverbal communication skills are influences by parent’s relational competence (Marschak, 1960; Stern, 2000; Knapp and Hall, 2009; Salo and Mäkelä, 2018).

#### 4.1.1 MIM-P specifics

Although significant correlations exist, it is essential to acknowledge the differences in the context of modest correlation coefficients for challenge and nurture (0.775**) and challenge and engagement (0.782**). These distinctions may stem from moderating variables impacting the connections between challenge, nurture, and engagement. For instance, participant age might moderate these relationships, resulting in varying correlation strengths across different age groups in the study. Additionally, the precision and reliability of measurements for challenge, nurture, and engagement can influence correlation strength as these aspects might be more difficult to rate as also suggested by the lower interrater reliability correlation. Measurement errors in these variables can attenuate observed correlations.

It is important to recognize that correlation coefficients primarily capture linear relationships between variables. If the relationships between challenge, nurture, and engagement are nonlinear, correlations may not fully convey their associations. Furthermore, it is crucial to remember that correlation does not imply causation. Causation is often more complex than simple correlations suggest. Further analysis and exploration may be necessary to gain a deeper understanding of the relationships between these dimensions within the MIM-P. As discussed in early prior investigations, parent’s mentalizing of their own upbringing holds significant importance in creating an atmosphere of shared intersubjectivity, particularly in the dimensions of engagement and challenge. This suggests that the quality of intersubjectivity between parent and child may be more contingent on the parent’s mentalizing capacity than on the child’s emotional development and competencies (Hart, 2018).

The lower interrater reliability for the “challenge” dimension can be attributed to several factors. The concept of “challenge” is inherently subjective and open to interpretation. Different raters may have varying perspectives on what constitutes a challenge, leading to greater disagreement in their assessments. The criteria for assessing the challenge dimension may be less clear than those for other dimensions, resulting in inconsistent ratings among different raters. Participants’ diverse experiences of challenges, influenced by factors like their background, expertise, or personal context, can hinder raters from reaching a consensus on challenge ratings. To improve reliability, it can be considered to provide clearer definitions and guidelines for assessing the challenge dimension to reduce ambiguity and ensure comprehensive training for raters to enhance their understanding and consistency in evaluating challenge.

#### 4.1.2 APCI specifics

The Nonverbal Communication Score has some of the same tendencies as the MIM-P Challenge Score. The correlation with Emotional Response (0.133*) show us how it is possible to have a low emotional response and a clear nonverbal communication in the interaction between primary caregiver and the child and to have a high emotional response and unclear emotional response which is also evident in the APCI profiles. However, it seems less likely to have the same reciprocal relationship between attunement and nonverbal communication (0.378**). The relationships between the APCI scores are nonlinear, so correlations may not fully convey their associations. However, it is worth noticing that the internal consistency has a much stronger correlation (0.78**) indicating that the combination of scores and the APCI profiles is what constitutes the reliability for APCI. As for the MIM-P, the precision and reliability of measurements for Nonverbal Communication Score can influence correlation strength as this aspect might be more difficult to rate as also suggested by the lower interrater reliability correlation. Looking at relevant literature, Knapp and Hall (2009) discussed how family communication environment can impact individual’s ability to both encode and decode nonverbal behavior. In families characterized by high expressiveness, children may excel in expressing themselves but might not develop refined decoding skills due to the clarity of surrounding expressions. Conversely, in families with lower expressivity, children may struggle with expression skills but excel in decoding because they need to interpret minimal or ambiguous cues from family members. As such, correlation does not imply causation and the results seem to confirm how nonverbal communication skills in a parent–child dyad in complex and non-linear.

The lower interrater reliability for the “non-verbal” score can be attributed to several factors. The Nonverbal Communication analysis does require some musical knowledge and skills and not all psychologist and pedagogues were equally skilled musically as this was not necessarily a part of their basic education leading to greater disagreement in their ratings. The definitions of clear and unclear turns and turn cycles may be less clear than the less musical focus on mutual attunement and emotional response. To improve reliability, it might make sense to provide clearer definitions and guidelines for assessing the non-verbal communication and through that ensure sufficient training for raters.

#### 4.1.3 Across APCI and MIM-P

The structure of the two tools is different in the way that all sub-scores in MIM-P strongly correlate indicating that the sub-scores are highly interdependent while the sub-scores in APCI correlate significantly but with a lower degree of correlation between scores while correlating stronger with the total score indicating that the scores are less interdependent while they all contribute to a coherent and cohesive construct. This aspect is also displayed in the correlation between the two tools where some scores highly correlate across the tools and others are further apart even though the total scores correlate strongly and significantly.

Both MIM-P and APCI measure the interaction between caregiver and child of a structured dyadic interaction. Where MIM-P is a play and activity-based method, the APCI is a tool based on nonverbal interaction using music. Both tools use video, and the interactions are scored through a recorded session. In general, the results indicate a high degree of correlation between the APCI and the MIM-P. The slightly lower correlation between Nonverbal Communication and Nurture and Challenge confirms the already discussed tendencies from the reliability results. The correlation between Mutual Attunement; Emotional Support; Parent–Child Interaction in the APCI profile and the five dimensions Structure; Co-regulation; Engagement; Nurture; Challenge in the MIM-P profile indicates substantial correlations but also that the tools seem to measure different aspects, which is not surprising as both tool focus on different but similar aspects of intersubjectivity and nonverbal interaction as well as social and emotional communication between a caregiver and a child.

### 4.2 Clinical applicability

Both the MIM-P and APCI are designed to be facilitated by a trained professional evaluating the caregiver’s and child’s interaction capacity. The aim of implementing MIM-P and APCI is to train professionals in tailoring realistic intervention plans to develop emotional, relational, and social competencies and set relevant goals and aims. The assessment tool requires one or two assessment sessions, which means it is not too demanding for the caregiver and child to take part. Also, most children find the assessment enjoyable, as many of the items consist of plays and music.

The MIM-P and APCI provide two structured ways of evaluating the intersubjectivity between caregiver and child. It is not a measure intended to stand alone, and it does not cover other critical areas, such as personality traits or cognitive abilities. However, with its focus on the caregiver and the child’s capacity to interact with and perform relevant activities together, the results can guide professionals on how to approach and support the child’s interaction capacity and through the intersubjective experiences and develop emotional capacities on both implicit and explicit levels.

The MIM-P and APCI may be helpful in organizing the intervention according to the resources and vulnerabilities in the caregiver-child’s interaction strategies based on assessment results with sources of validity and reliability assessment results. For instance, if the structure dimension is challenged an intervention aimed at helping the caregiver making structure for the child and helping the child accepting the caregiver’s structure is relevant etc. This might include working with structured play and games. If the attunement and the parent–child interaction are challenged an intervention working with rhythmic and synchronization activities through music therapy or “theraplay” are relevant, as the processes involved in these types of activities appear to improve the co-regulation dimension in the MIM-P and the attunement and interaction in the APCI etc. (Hart, 2016; Jacobsen and Holck, 2016; Daniel and Trevarthen, 2017; Jacobsen, 2017; Lindvang and Beck, 2017).

### 4.3 Limitations and further research

Several limitations of the present study are fully recognized. A larger, restrictive, and rigorous recruitment of non-referred participants would increase the validity of the results and enable investigations of norms of each tool and would make it possible to for instance perform exploratory factor analysis. However, this is not possible for the current sample because of the selection bias. We intend to evaluate factor analysis and confirmatory factor analysis in future studies with a sample that can be characterized as a general representation of the public. Furthermore, in the study there was an overrepresentation of female caregivers compared to male, which makes the psychometric analyses less trustworthy.

Another important limitation is the fact that it was not possible to blind the group of professional raters, as they knew when the participants were a referred or a non-referred dyad, which may have led to detection bias (Higgins et al., 2011).

The interdisciplinary inclusion of different professionals being trained and rating data might have made the psychometric analysis less valid, as the professions are not fully comparable even though all professionals went through the same certification process. In clinical practice, it is a great advantage to have interdisciplinary collaborations in using observation-based tools, but further analyses into differences across raters looking at professions are needed to better understand the depth of these clinical applications.

It would be relevant in future studies to compare with other standardized tools to further examine concurrent validity even though other former APCI and MIM-P studies have investigated this before. It would be pertinent to conduct an external validity study to juxtapose EAS with the APCI and MIM-P. This comparative analysis would enhance the robustness of all three observation-and attachment based assessment tools to measure dyadic caregiver-child relationship. To establish reliability norms for the MIM-P and APCI, future research should strive for a larger normative non-biased sample of caregiver-child dyads randomly recruited with no inclusion criteria for clinical or nonclinical features. Once establishment of norms has been investigated, novel studies for reliability and validity should be conducted. Further research may reveal whether the MIM-P as is indicated for APCI offers a suitable method for monitoring effect over time.

## 5 Conclusion

The empirical study of the psychometric properties of MIM-P and APCI revealed how the tools present sources of consistency, reliability, and validity of caregiver-child interaction capacity. There was a significant difference between scores from referred and non-referred groups and significant correlations between the observation-and interaction-based tools.

The results are promising both regarding the MIM-P and APCI. This study suggests that the MIM-P and APCI seems to offer a consistent measure of the caregiver-child intersubjectivity and is suited for preparing an intervention plan for either family therapy or intersubjectivity between professional and child, although more research is needed.

MIM-P and APCI both serve as powerful tools for the comprehensive assessment of the caregiver-child relationship. It delves into the overall quality and intrinsic nature of micro-regulation and sheds light on the strengths and vulnerabilities inherent in the nonverbal and emotional communication between the caregiver and the child, facilitating an in-depth examination of the intricate dynamics at play.

## Data availability statement

The raw data supporting the conclusions of this article will be made available by the authors, without undue reservation.

## Ethics statement

The studies involving humans were approved by SEKRETARIATET for DEN VIDENSKABSETISKE KOMITÉ for REGION NORDJYLLAND Niels Bohrs Vej 30 9220 Aalborg Ø. The studies were conducted in accordance with the local legislation and institutional requirements. Written informed consent for participation in this study was provided by the participants’ legal guardians/next of kin.

## Author contributions

SJ: Conceptualization, Data curation, Formal analysis, Investigation, Methodology, Project administration, Resources, Software, Supervision, Writing – original draft, Writing – review & editing. SH: Conceptualization, Funding acquisition, Investigation, Methodology, Project administration, Supervision, Writing – original draft, Writing – review & editing. JA-I: Data curation, Investigation, Methodology, Visualization, Writing – review & editing. GG: Conceptualization, Formal analysis, Investigation, Methodology, Writing – review & editing.
