# Supplementary material for: Psychometric properties of the Marschak Interaction Method of Psychometrics and the Assessment of Parent–Child Interaction within residential care and non-referred settings
Source: Front Psychol. 2024 Jan 8;14:1296113. doi: 10.3389/fpsyg.2023.1296113 (PMC10800955; doi:10.3389/fpsyg.2023.1296113)
Supplement: Supplementary file 1 [file Table_1.DOCX]

Supplementary Material

Article Title Validity and Reliability of the Marschak Interaction Method of Psychometrics (MIM-P) and the Assessment of Parent-Child Interaction (APCI) within Residential Care and Non-referred Settings

Stine L. Jacobsen1* †, Susan Hart1 †

*** Correspondence:** Corresponding Author: [slj@ikp.aau.dk](mailto:slj@ikp.aau.dk)

# Supplementary MIM-P details

**MIM-P activities**

Activity 1

3-5 years Adult and child each take one squeaky animal. Have the two animals play together.

6-10 years Adult and child take two figures and make them talk together.

11-17 years Adult and child take a set of figures each and make them talk together.

6-17 years Adult places 3 cotton balls on center of table. Adult and child stand at opposite ends of the table. Each takes a straw and tries to blow the cotton balls to other's side.

Activity 2

3-5 years Adult takes one bag of blocks. Hands other set of blocks to the child. Adult asks child to "Build one just like mine with your blocks." (5-8 blocks)

6-12 years Adult takes one bag of blocks. Hands other set of blocks to the child. Adult asks child to "Build one just like mine with your blocks." Afterwards adult ask the child to build one with his/her blocks for the adult to imitate. (10-15 blocks)

6-17 years Adult and child each take a piece of paper and a pen. Adult draws a quick drawing, encourages child to "Draw a drawing like mine."

Activity 3

3-12 years Adult and child put lotion on each other.

6-17 years Adult and child gives each other hand and/or back massage.

6-17 years Adult combs the child's hair and asks child to comb adult's hair.

13-17 years Adult and child look at each other's hands and read each other's fortune.

Activity 4

3-17 years Adult tells child about when child was a baby. Start like this: "When you were a little baby..."

3-17 years Adult tells child about when child was little. Start like this: "When you came to live with us..."

3-17 years Adult tells child about the time adult got to know child. Start like this: "When we got to know each other..."

Activity 5

3-17 years Adult teaches child something child doesn’t know.

Activity 6

3-17 years Adult leaves the room for one minute without child.

Activity 7

3-17 years Play a game that is familiar to both of you.

6-17 years Make a shared story. Start like this: "Now I'll start by telling the beginning of a story that you need to continue. Once you've come up with a few sentences, I'll continue. We'll keep going on until the story finishes."

13-17 years Adult asks child to describe a day in the child's life 10 years from now.

Activity 8

3-12 years Adult and child play three rounds of ‘Thumb wrestling’.

3-12 years Adult and child play a game of Mikado.

6-17 years Adult and child play three rounds of ‘Stones, scissors, paper’.

6-17 years Adult and child play a game of Tiddley-winks.

13-17 years Adult and child play three rounds of ‘Tic-tac-toe’.

13-17 years Adult and child play three rounds ‘Four in a row’.

Activity 9

3-12 years Adult and child put hats on each other.

3-12 years Adult and child put glasses on each other.

13-17 years Adult and child put hats and/or glasses on each other and create a dialogue in the different roles.

Activity 10

3-17 years Adult and child feed each.

**The five dimensions:**

Structure
The structural dimension shows whether the caregiver helps to delineate and clarify the child's experiences, ensures the child's safety, helps him/her to understand what is going on, and makes activities meaningful. It is about the caregiver's control and organizational skills, ways to give clear boundaries and about the ability to keep an agreed plan and abide by rules. In addition, the dimension is about the child's ability to accept boundaries, order, and the way to be given direction.

Co-regulation
The co-regulation dimension shows the synchronization capacity between caregiver and child. It is about the parent's ability to synchronize with the child and be indicative in relation to intuitive turn-taking, regulation, and rhythm, and about the child's ability to synchronize with the parent and be able to participate in turn-taking, be regulated and enter a rhythm. The synchronization consists of the regulated "choreography" between caregiver and child, which gives them a mutual experience of being on the same wavelength, e.g., through gaze exchanges, touch, voice melody, rhythm, etc.

Engagement
The engagement dimension shows the caregiver's ability to add excitement, surprise, and stimulation to maintain an optimal level of alertness and engagement in the child and ensure a shared engagement through play and fun. To support the child's development, these efforts must be commensurate with the child's level of development and emotional state. Dimension relates to the caregiver and the child's ability to play together so that it becomes fun and engaging and not just task-focused, and the child's ability to respond to, and be infected by the caregiver's engagement, interest, and joy.

Nurture
The nurture dimension is about the caregiver's ability to support the child's need for closeness and regulate and support the child when he/she is exhausted and stressed, in a way that is attuned to the child's needs. This dimension also relates to whether the child receives and enjoys the care in a natural way. It is not about the child's ability to provide care for the parent. It is about the caregiver's ability to respond to the child's need for care corresponding to his or her level of development and to the situation, and to respond in a calming and caring way to help the child cope with, for example, a stressful state. In addition, the child's ability to accept the caregiver's care and to seek comfort, care for himself, and regulate himself at an appropriate level of development is examined.

Challenge
The challenge dimension examines the caregiver's ability to stimulate the child's development, support exploration, encourage progress, exhibit appropriate expectations, find joy in the child's performance, and help the child accommodate any situations of loss. Thus, the challenge dimension is about the caregiver's ability to support and promote the child's efforts to master at a developmentally appropriate level by being curious and supporting the child's mastery in his/her approximate developmental zone and the child's ability to be able to/want to participate in activities that support and encourage efforts to make developmental progress.

The Scale


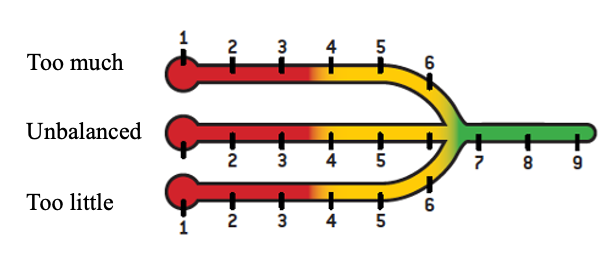


Supplementary Figure 1: GOOD, SOLID INTERACTION SKILLS, MASTERY AND RESOURCES,
INTERACTION SKILLS ARE PRESENT, BUT SHOULD BE SUPPORTED, INTERACTION SKILLS ARE DEFICIENT

# Supplementary APCI details

**Attunement Analysis**

Step-by-step guide

1. Watch and listen to the entire clip/exercise. If helpful, write the clip time in the box at the beginning.
2. Consider each question one at a time for each exercise and assess the alignment of the child's and parent's leading and following behaviours from 1-9 using the definitions described below.
3. Next, consider the dyad's need for support along the way using the definitions below, rating from 1-9.
4. Finally, consider child autonomy and use the definitions below and rate from 1-7.

To what extent does the **child** take the initiative to lead? 1-9

Not attuned 1-3

In this category, the child pays little or no attention to the other person, which in its simplicity makes attunement impossible. The child is in their own world with no focus on leading, or may not be looking for the other person to lead them. The child may also take constant initiative without regard or interest in the other person's reaction or behaviour.

Not consistent 4-6

Here, the child will tend to go in and out of their own world and may sometimes try to

However, not always with attention to the other person's reaction. Perhaps the child in

able to sometimes be aligned in its initiatives to lead with minimal attention to

the other party's reaction and behaviour.

Attuned 7-9

Here, the child is balanced in their initiatives and takes the space with consideration for the other

person. The child can consistently and naturally pay attention to the other person, even without the other person paying attention the other way round. An equal exchange is about the dyad equally sharing space and taking turns equally leading the other and taking the initiative. When one leader follows the other.

To what extent does the **child** follow the parent's initiatives? 1-9

Not attuned 1-3

In this category, the child pays little or no attention to the other, which in its simplicity makes attunement impossible. The child is in their own world with no focus on following, or may not seek the other person in terms of following them. The child may also be deeply dependent on the other without being able to react without initiative from the other.

Not consistent 4-6

Here, the child will tend to go in and out of their own world and may sometimes try to

follow the other person, but not always with attention to whether the other person is registering it. Perhaps the child is sometimes able to be attuned in their intentions to follow with minimal attention to the other person's reaction and behaviour.

Attuned 7-9

Here, the child is aligned in their intention to follow and makes room for the other person's initiatives. The child can consistently and naturally pay attention to the other person without the other person paying attention in the opposite direction. An equal exchange is about the dyad sharing space in an equal way and taking turns equally following the other and letting the other decide. When one leader follows the other.

To what extent does the **parent/carer** take the initiative to lead? 1-9

Not tuned 1-3

In this category, the parent/carer pays little or no attention to the other, which in its simplicity makes attunement impossible. The adult is in their own world with no focus on leading, or may not be looking for the other person to lead them. The parent/caregiver may also take constant initiative without regard for the other party or interest in the other party's reaction or behaviour.

Not consistent 4-6

Here, the adult will tend to go in and out of their own world, and maybe sometimes

try to lead the other person, but not always with attention to the other person's reaction. Perhaps the parent/educator is sometimes able to be balanced in their initiatives to lead with minimal attention to the other person's reaction and behaviour.

Attuned 7-9

Here, the parent/carer is balanced in their initiatives and takes the space with consideration for the other person. The adult can consistently and naturally pay attention to the other person without the other person looking the other way. An equal exchange is about the dyad sharing the space in an equal way and taking turns equally leading the other and taking the initiative. When one leader follows the other.

To what extent does the **parent/carer** follow the child's initiatives? 1-9

Not tuned 1-3

In this category, the parent or carer pays little or no attention to the other person, which in its simplicity makes attunement impossible. The adult is in their own world with no focus on following, or perhaps not seeking the other person in terms of following them. The parent/carer may also be deeply dependent on the other without being able to react without initiative from the other.

Not consistent 4-6

Here, the adult will tend to go in and out of their own world and perhaps sometimes try to follow the other person, although not always with attention to whether the other person is registering it. Perhaps the parent/educator is sometimes able to be balanced in their intentions to follow with minimal attention to the other person's reaction and behaviour.

Attuned 7-9

Here, the parent/carer is aligned in their intention to follow and makes room for the other person's initiatives. The adult can consistently and naturally pay attention to the other person without the other person paying attention in the opposite direction. An equal exchange is about the dyad sharing the space in an equal way and taking turns equally following the other and letting the other decide. When one leader follows the other.

**Non-verbal Communication Analysis**

To assess how the dyad produces and reads each other's non-verbal signals, the turn-taking exercise - exercise 2 - is analyzed. There is equal focus on turn taking and turn giving in this analysis.

Step-by-step guide

1. Watch and listen to the full activity (or a two-minute clip). If useful, you can add the clip time in the box on the left.
2. When you watch the clip again, add a tick every time the child or adult has a turn mark and stop the clip
3. Next, assess whether the participant is passing on their turn - tick
4. Now assess whether the participant uses clear signs to convey the trip, if it is unclear or if the trip is conveyed in a confusing way - tick. Definitions of types of turn-taking are listed below.
5. Trip cycle type - how many trips were clean? A trip cycle consists of the following: A plays and stops, B plays and stops. If either A or B is interrupted, the trip cycle is interrupted. If no one is interrupted, the turn cycle is clean. Write the totals in the box.

Defining types of clear turn-givings

*Clear turn-givings* can be when a participant gives a musical ending to their turn as a signal that the turn is being passed on or uses their body language to indicate that a turn is being passed on. For example:

- A *melodic* ending has an upward or downward pattern that forms a clear cadence in the harmony
- A *dynamic* walk can have a sudden increase in momentum or a gradual decrease in momentum, as if the participant is *fading out*
- A *rhythmic* ending will be a clear piece that sounds complete.
- *Eye contact* - one participant can look at the other, or their instrument, to offer the ride
- *Hands* - a participant can point at the other person's instrument or towards them with their hands or their own instrument
- *Smile* - the ride is offered to the other participant with a smile
- *Nik* - an instructive way to pass on the ride.

*Confusing turn-taking* is when one participant sends mixed signals to the other, for example, combining signs of turn-taking with signs of wanting to continue playing, or the turn is given to a third person (the therapist). Without the following being considered an exhaustive list, these options may include:

- The participant looks at the other (gestural turn-taking), but continues to play or move their instrument
- Nods to the other participant but looks in a different direction
- Smiles, looks, nods to the other participant, but continues to play.

This type of turn-taking will often create a slight pause or awkwardness in the game while the other participant thinks about how to react.

**Emotional Response Analysis**

The final part of the APCI analysis deals with the parent's emotional response to the child. The score is derived from almost all activities and is analysed by re-watching the clips to identify the parent's response. It is important to consider only the parent's response to the child. The response will include verbal and non-verbal behaviour. These behaviours can be in the style and content of the music, conversations or outbursts from the parent or child, body language such as leaning away from or towards each other or their eye contact, smiles and laughter. All of these actions can be categorised as part of six response types (see below). It's your role to determine how evident the response types are in the session.

Levels of response

0 - Not clear at any point
1 - Slightly obvious at few or many times
2 - Quite evident at few or many times

Step-by-step guide

1. Watch and listen to the clip from the beginning (when the family looks around the room while "preparing")
2. When you watch the clip again, consider each response type (definitions below). Give the level of response type a score on the data sheet (either 0, 1 or 2)
3. Repeat step 2 for exercises 1, 3 and 4
4. Add the columns together and record the total in the last box

**Definitions of emotional response types**

Rejecting
This category refers to insecure attachment behaviour where the child expects to be rejected by the parent/caregiver, who may even enjoy leaving the child unfulfilled. The adult may struggle to relate to the child and may completely withhold their emotions. The parent/carer may ignore the child when the child seeks attention.

Dominating
This refers to a parent/carer who prioritises their own needs over the child's. He or she may be dominant, autocratic and perhaps even impulse-driven. In the session, the adult may deliberately dominate the interaction with the child because of their own need to control.

Over-involved
This parent/carer may interrupt the child or ask them to perform tasks that are too difficult for their developmental age. The adult may be too intrusive, pushy and worried. He or she may not allow the child to be independent and dictate how or whether to make choices or express themselves.

Passive
The parent/carer in this category rarely responds to their child and may have difficulty reading and understanding non-verbal cues. This type of parent struggles to stimulate their child, which can result in a mutually passive interaction. The adult may be inattentive to their child's needs.

Supportive
Here, the adult has a positive engagement with the child. He or she adapts his or her behaviour towards the child to regain and maintain the child's interest. The parent/carer protects the child and helps them develop a healthy sense of independence. During the session, the parent will support the child through involvement, recognition and guidance.

Emotionally exchanging
This category refers to affect matching and intersubjective exchange of emotions between parent and child. Here, the adult will respond in accordance with their own feelings and there will be an obvious emotional connection in the dyad. During the session, the parent will respond to the child's emotional expression by expressing their own emotions and sharing them with the child (both positive and negative emotions, but in an appropriate way).

**APCI Profile examples**

MCSI: The APCI profile indicates that the primary caregiver and child are functioning well when it comes to being attentive and attuned towards each other. The parent and child are successfully communicating nonverbally both in producing clear signals and understanding each other’s signals.

| M -  Mutually attuned | There were clear musical conversations and interactions between the dyad showing the parent/carer and child are well attuned. For example, if the child played a music motif, the parent responded appropriately, matching tempi, dynamics or intensity. The parent and child are communicating in an effective and meaningful way. |
| --- | --- |
| C -  Clear nonverbal communication | The parent/carer and child are successfully communicating in a nonverbal way. They understand each-others signals and can respond in a way that matches the dynamic of the other, ensuring the message being sent is understood. |
| S -  Supportive parenting | The parent/carer understands the needs of the child and offers encouragement when the child needs it. For example, if the child is cautious when entering the therapy space, the parent will offer verbal assistance or change their body language to encourage the child to feel safe. |
| I -  Independent child | The child feels comfortable in leading the activities and this is reflected in its ability to take the lead in the music. The child can explore and experiment during activities. The child often has its own opinions and can voice ideas and concerns in an age appropriate way. |

NULD: The APCI profile indicates that the primary caregiver and child are not functioning well when it comes to being attentive and attuned towards each other. The parent/carer and child are not successfully communicating nonverbally and are not producing clear enough signals and/or the individuals are not able to understand and read the signals of the other.

| N -  Non Mutually Attuned | The dyad is not able to consistently be attentive to each other or attuned to the other. The parent/carer and child may not be listening or responding to one another in a useful way either due to unclear signals and musical expressions or sometimes lack of capacity to focus on the need of the other. |
| --- | --- |
| U -  Unclear nonverbal communication | The parent and child are challenged in their nonverbal communication and successful in this type of interaction. This means they are struggling to encode prosody, body posture and facial expressions into their communication and/or they are unable to understand (decode) these messages from the other. The parent/carer and child can be experiencing difficulty in both encoding and decoding, resulting in mismatched ‘answers’ to nonverbal signals. This confusion often results in the parent and child feeling frustrated and/or taking a passive stance – not expecting to be heard. However, if the child is 6 or younger the nonverbal communication skills might not naturally be fully developed yet. |
| L - Lack of Parenting Support | The parent/carer is not consistently emotionally supportive of the child and this is shown through a lack of attendance to their needs. This can be shown through lack of verbal and nonverbal interaction and/or withdrawal from emotional engagement with the child. Often lack of support is accompanied with passive parental responses or focus on the need of the parent/carer in the parent (and the child). |
| D -  Dependent child | The child feels uncomfortable in leading the activities and this is reflected in its reluctance or inability to take the lead in the music. The child might be less inclined to explore and experiment during activities. The child might reply on the lead of the parent or the therapist. |
